# Successful treatment of glaucoma in Sturge–Weber syndrome using PreserFlo™ microshunt with intraluminal stenting: a case report

**DOI:** 10.1016/j.ajoc.2025.102440

**Published:** 2025-09-26

**Authors:** Hidekazu Inami, Ryo Tomita, Kenya Yuki

**Affiliations:** Department of Ophthalmology, Nagoya University Graduate School of Medicine, 65, Tsurumai, Showa ward, Nagoya, Aichi, 466-8560, Japan

**Keywords:** Sturge–Weber syndrome, PreserFlo™ microshunt, Glaucoma, Intraocular pressure

## Abstract

**Purpose:**

We report a case of glaucoma associated with Sturge–Weber syndrome (SWS) that was successfully managed with PreserFlo™ microshunt (PFM) insertion combined with two intraluminal 10-0 nylon suture stents.

**Observations:**

A 27-year-old female patient with SWS and persistently elevated intraocular pressure (IOP) in the right eye was referred to our hospital for surgical treatment. The patient was diagnosed with bilateral SWS-related glaucoma. She first underwent a right trabeculotomy at the age of 11, followed by a trabeculectomy on the same eye. At the age of 13, a trabeculectomy was performed on the left eye. At the time of presentation, her right eye IOP was 28 mmHg. A PFM with two 10-0 nylon intraluminal sutures was implanted. On postoperative day 1, IOP decreased to 16 mmHg. The shunt was well positioned without complications. On day 17, IOP rose to 20 mmHg, leading to the removal of one suture. On day 22, IOP dropped to 18 mmHg, and the second suture was removed.

**Conclusions:**

Herein, we successfully executed PFM insertion with intraluminal stenting in a patient with SWS, resulting in effective IOP reduction without any serious postoperative complications. These findings suggest that PFM insertion with stenting could be a viable treatment option for glaucoma associated with SWS.

## Highlights

• Glaucoma associated with SWS frequently necessitates surgical management.
• PFM insertion with stenting is beneficial for minimizing postoperative hypotony.
• PFM insertion with stenting may be an effective strategy for glaucoma associated with SWS.

## 1 Introduction

Sturge–Weber syndrome (SWS) is a nonhereditary congenital disorder that characterized by leptomeningeal hemangioma, facial angiokeratoma, port-wine stains, and various ocular abnormalities.^1^ It is estimated to affect one in every 20,000–50,000 people with no racial bias and an equal distribution among men and women. SWS is the third most common neurocutaneous syndrome after neurofibromatosis and tuberous sclerosis.^1^^,^^2^ Glaucoma is the most common ocular manifestation of SWS, and it can be congenital or develop later in life.^3^ Approximately 60 % of SWS glaucoma cases develop within the first two years of life, with the remaining 40 % occurring in childhood or adulthood.^3^ The exact pathophysiology is still unknown but is believed that it stems from abnormal anterior chamber angle development.^4^ Other proposed mechanisms include elevated episcleral venous pressure, which is prevalent among older children and young adults, as well as impaired aqueous drainage caused by distal outflow resistance.^3^^,^^5^

Glaucoma is the most common ocular manifestations in SWS, with glaucoma occurring in approximately 30 %–70 % of cases.^6^ Medical therapy is usually the first-line treatment for glaucoma caused by SWS; however, surgical intervention is necessary in most cases.^5^ The PreserFlo™ microshunt (PFM; Santen, Osaka, Japan) is an 8.5 mm glaucoma drainage device composed of poly (styrene-block-isobutylene-block-styrene; SIBS), a biocompatible and bioinert polymer.^7^ PFM insertion with mitomycin C (MMC) is considered to be effective in lowering intraocular pressure and is linked to a lower incidence of hypotony than trabeculectomy with MMC.^8^

In this report, we describe a case of SWS-associated glaucoma that was successfully treated with PFM insertion and intraluminal stenting to prevent postoperative hypotony and achieve effective IOP control.

## 2 Case report

A 27-year-old female was referred to our hospital for surgical treatment of a persistently elevated right eye IOP. Congenital glaucoma was identified after she was diagnosed with SWS postnatally. She first underwent a trabeculotomy in the right eye at the age of 11; however, due to inadequate IOP control, a trabeculectomy was subsequently performed in the same eye. At the age of 13, she underwent a trabeculectomy in the left eye.

At the initial visit, the patient's best-corrected visual acuity (BCVA) was assessed using a decimal visual acuity chart and converted to the corresponding logarithm of the minimum angle of resolution (logMAR) values for both eyes. Refraction revealed a refractive error of a −9.00 diopters sphere with a −2.25 diopter cylinder at 20° in the right eye and a −3.50 diopter sphere with a −4.00 diopter cylinder at 180° in the left eye. The IOP determined with Goldman applanation tonometry (GAT) was 28 mmHg and 11 mmHg in the right and left eyes, respectively. Specular microscopy revealed a central corneal thickness of 490 μm in the right eye and 514 μm in the left eye. The anterior chamber, lens, and vitreous were unremarkable in both eyes. The patient exhibited bilateral facial hemangiomas located along the distribution of the second trigeminal nerve. The fundus examination revealed glaucomatous optic disc cupping, with cup to disc ratios of 0.9 and 0.7 in the right and left eyes, respectively. A choroidal hemangioma was observed in the right eye but not in the left. Goldmann perimetry revealed a nasal step and paracentral scotoma in the right eye visual field, whereas no significant visual field defects were observed in the left eye. Gonioscopy of the right eye demonstrated inferior angle pigmentation and an open anterior chamber angle, graded as Shaffer classification grade 4. The left eye also exhibited an open angle, graded as Shaffer classification grade 4, without significant pigmentation. The nasal-superior conjunctiva of the right eye exhibited satisfactory mobility. Postoperative conjunctival scarring from trabeculectomy was observed at the 5 o'clock position in the right eye and the 12 o'clock position in the left eye. The patient's right eye received therapy with 0.03 % bimatoprost, 1 % brinzolamide tartrate, 0.5 % timolol maleate, 0.4 % ripasudil hydrochloride hydrate, 0.1 % brimonidine tartrate, and 0.01 % bunazosin hydrochloride eye drops, as well as one 250 mg acetazolamide tablet per day. The IOP, however, remained consistently elevated, at 28 mmHg.

**Fig. 1:**
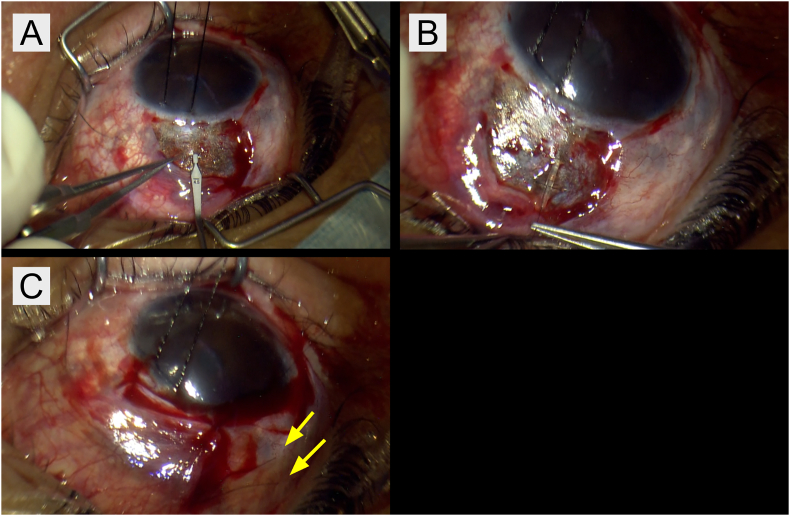
Fig. 1A–C. Intraoperative photographs illustrating the surgical technique. (A) Creation of a scleral pocket utilizing a double-step knife. (B) Placement of a 10-0 nylon suture within the PFM lumen. (C) Externalization of the posterior ends of two stent sutures onto the conjunctival surface through a vertical conjunctival incision. The two yellow arrows indicate the 10-0 nylon sutures in the image. (For interpretation of the references to colour in this figure legend, the reader is referred to the Web version of this article.)

A stand-alone PFM implantation was performed on the right eye under sub-Tenon's anesthesia using 2 % xylocaine. Following a vertical conjunctival incision at the 12 o'clock position, a limbal incision was made, and the superonasal conjunctiva was dissected from the sclera. After performing a superonasal conjunctival peritomy, 0.04 % MMC was applied beneath the conjunctival flap using a sponge and subsequently irrigated after 3 min. Using a double-step knife provided in the kit, a scleral pocket was created 3 mm posterior to the corneal limbus (Fig. 1A). The double-step knife was advanced horizontally for 2 mm through the sclera from the entry site and then angled anteriorly to enter the anterior chamber in a plane parallel to the iris. The PFM was carefully inserted through the scleral pocket, and two 10-0 nylon sutures were secured in the shunt tube (Fig. 1B). The posterior ends of the 10-0 nylon sutures were externalized onto the conjunctival surface through a vertical conjunctival incision (Fig. 1C). Finally, proper shunt positioning was confirmed, and no evidence of bleb leakage was observed.

**Fig. 2:**
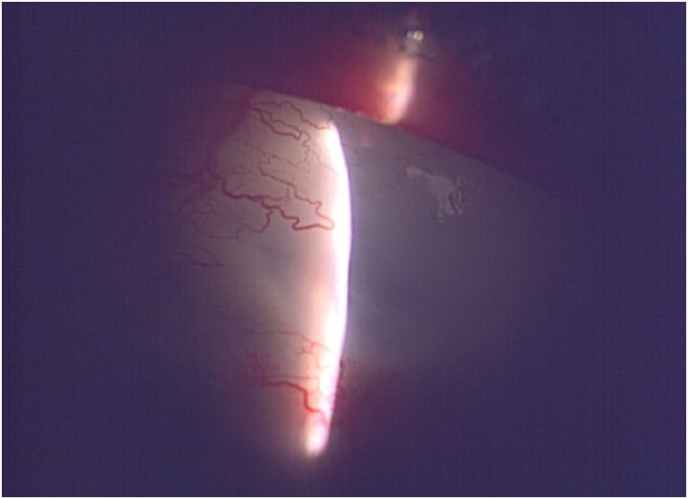
Slit-lamp photograph at three months postoperatively The bleb appeared elevated, and the IOP was 14 mmHg.

**Fig. 3:**
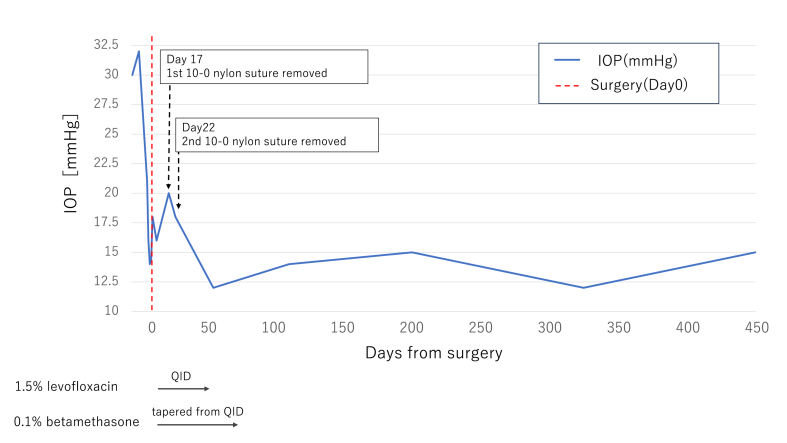
Variations in intraocular pressure (IOP) before and after surgery, and postoperative use of antiglaucoma medications. The first intraluminal 10-0 nylon suture was removed on postoperative day 17, followed by removal of the end suture on day 22. Postoperatively, 1.5 % levofloxacin was administered four times daily, and 0.1 % betamethasone was initially given four times daily with gradual tapering. IOP was monitored over a 15-month postoperative period.

Postoperative treatment included 1.5 % levofloxacin and 0.1 % betamethasone eye drops, administered four times daily for 50 days and 80 days, respectively. On the first postoperative day, the right eye's IOP measured by GAT, was 16 mmHg. PFM was correctly positioned in the anterior chamber, with no signs of choroidal detachment or hemorrhage. Throughout the follow-up period, IOP remained stable at 14–18 mmHg. On postoperative day 17, IOP rose to 20 mmHg, necessitating the removal of one stent. On postoperative day 22, IOP was 18 mmHg in the right eye, and bleb morphology was favorable, prompting the removal of the second stent. Three months after surgery, IOP was 14 mmHg without glaucoma medication, and the bleb was large (Fig. 2). At 15 months after surgery, visual acuity in the right eye was 0.2 logMAR, and IOP was 15 mmHg without medication (Fig. 3).

## 3 Discussion

Managing SWS-associated glaucoma is difficult, necessitating an individualized treatment plan. Goniotomy and trabeculotomy are frequently used as primary surgical options for congenital glaucoma, including early-onset glaucoma in SWS because the anterior chamber morphology is similar to that of primary congenital glaucoma.^9^ Previous studies have revealed that the long-term prognosis of angle surgeries in glaucoma associated with SWS is limited, with primary goniotomy maintaining stability for only 6–12 months and primary trabeculotomy achieving a slightly longer mean duration of 21 months.^10^ Given the limited long-term efficacy of trabeculotomy in patients with SWS, trabeculectomy or glaucoma drainage devices may offer relatively superior IOP control.^7^ Agarwal et al. reported that 61.1 % of eyes with SWS-associated glaucoma achieved IOP <22 mmHg after combined trabeculotomy-trabeculectomy (mean follow-up 42 months),^11^ while Sarker et al. found 70 % of eyes maintained IOP <21 mmHg at 24 months after trabeculectomy.^12^ In children with SWS, two-stage Baerveldt glaucoma implant surgery achieved IOP ≤21 mmHg over a mean follow-up of 35 months with minimal complications.^13^

However, postoperative hypotony-related complications are notably more common in eyes with SWS. In patients undergoing glaucoma surgery, early postoperative choroidal effusion has been reported in approximately 13 % of eyes following trabeculectomy and 14 % after glaucoma drainage device implantation.^14^ However, in patients with SWS, the incidence appears to be significantly higher. For example, Keverline and Hiles reported choroidal effusions in 24 % of eyes with SWS-associated glaucoma following trabeculectomy.^15^ Similarly, Kocyła-Karczmarewicz et al. documented post-operative complications in a cohort of nine eyes with SWS-associated glaucoma undergoing trabeculectomy during childhood, with choroidal effusion in Also, the Paul Glaucoma Implant has smaller lumen than BGI and can be tied externally to reduce risk of hypotony on these cases with good long term outcomes. It is described as a part of the this RCT 56 % and hypotony in 33 % of eyes.^16^ Furthermore, in a retrospective review of 22 eyes with pediatric SWS-associated glaucoma treated with glaucoma drainage devices, postoperative hypotony developed in 18 % of eyes.^17^ These results emphasize the elevated risk of hypotony-related complications in eyes with SWS following intraocular surgery compared to the general glaucoma cohort.

To address these challenges, newer devices with flow-restricting designs have been developed. The Paul Glaucoma Implant, which has a smaller lumen and allows external ligation, has been shown to reduce the risk of hypotony with favorable long-term outcomes in a recent randomized trial.^18^ The Paul GIaucoma Implant may represent a promising option in such high-risk cases. In this setting, PFM implantation may offer a safer option, as described in the present report. A prospective, randomized multicenter trial demonstrated that PFM implantation with MMC achieved comparable IOP reduction to trabeculectomy with MMC, while being associated with fewer hypotony events.^8^ In addition, a retrospective case-control study of 42 highly myopic primary open-angle glaucoma (POAG) eyes found that intraluminal stenting reduced the incidence of early postoperative hypotony from 29 % to 5 % without compromising overall surgical success.^19^ They concluded that PFM intraluminal stenting is an effective strategy for preventing early postoperative hypotony in patients with POAG and high myopia. In previously reported pediatric cases of Sturge–Weber syndrome (aged 0.6–9.4 years) undergoing PFM implantation, four out of five patients achieved favorable IOP reduction during follow-up (up to approximately one year), and no severe hypotony was observed.^20^, ^21^, ^22^ These findings suggest that PFM surgery may be a viable option for managing glaucoma in adult patients with SWS, with stenting potentially helping to reduce the risk of postoperative hypotony in those with complex vascular abnormalities.

In the present case, the decision to employ PFM with intraluminal stenting was guided by several patient-specific factors. Considering the patient's young age and history of prior failed filtering surgeries, we aimed to adopt a less invasive and safer surgical approach. Additionally, given the elevated risk of postoperative complications such as hypotony and choroidal effusion frequently noted in eyes with SWS, we selected the PFM with intraluminal stenting to mitigate the risk of hypotony while ensuring adequate IOP control. At the 15-month postoperative follow-up, intraocular pressure remained stable without requiring antiglaucoma medications, and no adverse events were noted. These findings indicate that PFM implantation with adjunctive stenting may be a promising surgical option for treating SWS-associated glaucoma. However, additional cases and long-term follow-up are required to comprehensively assess its safety and effectiveness. Furthermore, patient age, demographic characteristics, and underlying SWS pathophysiology likely impact both surgical outcomes and procedure selection. Continued investigation into these factors will be essential to refine individualized treatment approaches.

## CRediT authorship contribution statement

**Hidekazu Inami:** Writing – original draft, Conceptualization. **Ryo Tomita:** Writing – review & editing, Supervision, Funding acquisition. **Kenya Yuki:** Writing – review & editing, Supervision, Project administration, Formal analysis, Data curation, Conceptualization.

## Patient consent

Consent to publish this case report has been obtained from the patient in writing. This report does not contain any personal identifying information.

## Funding

This study was supported by Grant-in-Aid for Young Scientists (RT, grant no. 21K16870) from JSPS KAKENHI (http://www.jsps.go.jp/), Suda Memorial Glaucoma research grant (RT) and the Japan Glaucoma Society Research Project Support Program (RT).

## Declaration of competing interest

The authors declare that they have no known competing financial interests or personal relationships that could have appeared to influence the work reported in this paper.

## References

[1] Higueros E., Roe E., Granell E., Baselga E. (2017). Sturge-Weber syndrome: a review. Actas Dermosifiliogr.

[2] Sudarsanam A., Ardern-Holmes S.L. (2013). Sturge-Weber syndrome: from the past to the present. Eur J Paediatr Neurol.

[3] Alejandro J., Luat A.F., Juhász C. (2018). A multidisciplinary consensus for clinical care and research needs for Sturge-Weber syndrome. Pediatr Neurol.

[4] Thorpe J., Frelin L., McCann M. (2021). Identification of a mosaic activating mutation in GNA11 in atypical Sturge-Weber syndrome. J Invest Dermatol.

[5] Hassanpour K K., Nourinia R R., Gerami E E., Mahmoudi G G., Esfandiari H H. (2021). Ocular manifestations of the Sturge-Weber syndrome. J Ophthalmic Vis Res.

[6] ComiA (2007). Update on Sturge-Weber syndrome: diagnosis, treatment, quantitative measures, and controversies. Lymphatic Res Biol.

[7] Gambini G., Carlà M.M., Giannuzzi F. (2022). PreserFlo® microshunt: an overview of this minimally invasive device for open-angle glaucoma. Vision (Basel).

[8] Panarelli J.F., Moster M.R., Garcia-Feijoo J. (2024). Ab-externo MicroShunt versus trabeculectomy in primary open-angle glaucoma: two-year results from a randomized, multicenter study. Ophthalmology.

[9] Olsen K.E., Huang A.S., Wright M.M. (1998). The efficacy of goniotomy/trabeculotomy in early-onset glaucoma associated with Sturge-Weber syndrome. J AAPOS.

[10] Patrianakos T.D., Nagao K., Walton D.S. (2008). Surgical management of glaucoma with the Sturge-Weber syndrome. Int Ophthalmol Clin.

[11] Agarwal H.C., Sandramouli S., Sihota R., Sood N.N. (1993). Sturge-Weber syndrome: management of glaucoma with combined trabeculotomy-trabeculectomy. Ophthalmic Surg.

[12] Sarker B.K., Malek M.A., Mannaf S.M. (2021). Outcome of trabeculectomy versus Ahmed glaucoma valve implantation in the surgical management of glaucoma in patients with Sturge-Weber syndrome. Br J Ophthalmol.

[13] Budenz D.L., Sakamoto D., Eliezer R., Varma R., Heuer D.K. (2000). Two-staged Baerveldt glaucoma implant for childhood glaucoma associated with Sturge-Weber syndrome. Ophthalmology.

[14] Gedde S.J., Schiffman J.C., Feuer W.J., Herndon L.W., Brandt J.D., Budenz D.L. (2009). Tube versus trabeculectomy study group, three-year follow-up of the tube versus trabeculectomy study. Am J Ophthalmol.

[15] Keverline P.O., Hiles D.A. (1976). Trabeculectomy for adolescent-onset glaucoma in the Sturge-Weber syndrome. J Pediatr Ophthalmol.

[16] Kocyła-Karczmarewicz B., Klimczak-Slaczka D., Grałek M., Chipczyńska B. (2006). Childhood glaucoma associated with Sturge-Weber syndrome – the efficacy of cyclophotocoagulation and other therapeutic methods. Klin Oczna.

[17] Glaser T.S., Meekins L.C., Freedman S.F. (2021). Outcomes and lessons learned from two decades' experience with glaucoma drainage device implantation for refractory Sturge-Weber–associated childhood glaucoma. J AAPOS.

[18] Elhusseiny M., Khaled O.M., Chauhan M.Z., Sayed M.S., Shaarawy T. (2025). Initial results of the Paul Ahmed Comparison (PAC) study in refractory childhood glaucoma. Am J Ophthalmol.

[19] Lupardi E., Laffi G.L., Moramarco A., Barboni P., Fontana L. (2023). Systematic PreserFlo® microshunt intraluminal stenting for hypotony prevention in highly myopic patients: a comparative study. J Clin Med.

[20] Sesma G., AlHijji L., AlRomaih A., Schargel Kint (2025). Innovative use of PreserFlo microshunt in an infant with Sturge-Weber syndrome-related glaucoma. J Surg Case Rep.

[21] Tanaka Y., Kasahara S., Tomita R., Nonobe N., Kawase K., Yuki K. (2025). Preserflo™ microshunt for the treatment of refractory childhood glaucoma: a case series study. Am J Ophthalmol Case Rep.

[22] Brandt J.D. (2022). Use of a novel microshunt in refractory childhood glaucoma: initial experience in a compassionate use/early access cohort. Am J Ophthalmol.

